# Implementation of a Mobile Platform Based on Fiber Bragg Grating Sensors for Automotive Traffic Monitoring

**DOI:** 10.3390/s20061567

**Published:** 2020-03-11

**Authors:** Kivilcim Yuksel, Damien Kinet, Karima Chah, Christophe Caucheteur

**Affiliations:** 1 Electronics Engineering Department, Izmir Institute of Technology, TR-35430 Urla, Izmir, Turkey; kivilcimyuksel@iyte.edu.tr; 2 Electromagnetism and Telecommunication Department, Faculty of Engineering, University of Mons, Boulevard Dolez 31, 7000 Mons, Belgium; damien.kinet@umons.ac.be (D.K.); Karima.Chah@umons.ac.be (K.C.)

**Keywords:** fiber optic sensors, FBG, traffic monitoring, smart-city

## Abstract

Instrumentation techniques, implementation and installation methods are major concerns in today’s distributed and quasi-distributed monitoring applications using fiber optic sensors. Although many successful traffic monitoring experiments have been reported using Fiber Bragg Gratings (FBGs), there has been no standardized solution proposed so far to have FBG seamlessly implemented in roads. In this work, we investigate a mobile platform including FBG sensors that can be positioned on roads for the purpose of vehicle speed measurements. The experimental results prove the efficiency of the proposed platform, providing a perspective toward weigh-in-motion systems.

## 1. Introduction

Today’s technology companies, entrepreneurs, and local governments have been focusing on the phenomenon of “smart city” in the focus of attention for the purpose of improving the quality of life for citizens [1]. One of the most important implications of this approach in operating, monitoring, and assessment systems is the integration of fiber optic sensors into smart-city infrastructures.

In this context, Fiber Bragg Gratings (FBGs) have been shown as capable of being the nervous system of infrastructures due to the many advantages of these mass-producible intrinsic sensing devices. The advantages include their inherent wavelength-encoded demodulation feature, resistance to electromagnetic interference, great configurability with multiplexing capability (several tens of cascaded sensors at different wavelengths within a single optical fiber can be interrogated using only one piece of equipment), remote operation, passive and lightning/corrosion-resistive nature, as well as their small size.

Through real-time field trials, usage of FBG sensors has already been demonstrated as a feasible solution for traffic surveillance in railways [2,3], damage detection of highway bridges under traffic flow [4], monitoring old tunnels, weighing vehicles [5,6], and asphalt pavement analysis and design [1,7].

The most crucial point in almost all of the above-mentioned outside field attempts to perform Experimental measurements with FBG sensors is the instrumentation design and installation method. Regarding this aspect, there are many parameters to consider that will inevitably affect the final result. Mechanical properties and dimensions of the measurement platform, distance between the sensor fiber and the road, material used for sensor protection and coating, number of cascaded sensor points, distance between sensors, gauge length, and the type of optical fiber used for sensor inscription are only some of those parameters. There has so far been no standardized design recipe or commercial product of FBG-based traffic monitoring system that can be seamlessly implemented in roads under traffic. Particularly, the proper long-term calibration of a sensor system to monitor the differences in measured and real values of the target physical parameter (e.g., strain, speed) is a challenging issue.

As a result of these test and calibration problems, there remains ample room for more experimental investigations of different design approaches. In order to experiment more on the outside field requirements of traffic monitoring, in this work, we implemented a mobile platform including FBG sensors that can be positioned on the road, together with an interrogation unit (at a remote location) and a signal processing tool. Two measurement campaigns were conducted, one on a road during its normal operation and the other on another road closed to traffic. Through these preliminary proof-of-concept measurements, we successfully demonstrated the feasibility of the proposed platform for wheel tracing and vehicle speed determination. The roadmaps for a weigh-in-motion measurement system have been discussed as a future perspective.

## 2. Instrumentation and Road Installation

**Figure 1:**
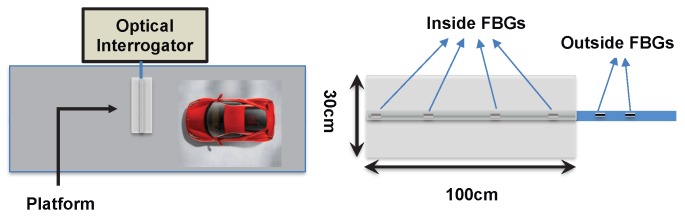
Typical installation layout.

As sketched in Figure 1, the measurement setup consisted of an optical interrogator and a platform containing an optical fiber with the FBG sensors. The bottom plate of the platform was made from smooth steel, while the top of the plate contained an aluminum profile that protects FBGs and adhesives. The plate and profile were taped using silver cloth tape. They were also glued together using a two-component polyurethane adhesive. Inside of the profile, there was a blue reinforced furcation tube covering the optical fiber containing 4 FBGs. A small portion of the tube including 2 additional FBGs lay outside of the platform. The FBGs, all cascaded on the same optical fiber, were about 25 cm apart from each other. During the measurements, the platform (containing 4 FBGs) was placed on the road while the rest of the furcation tube (containing 2 FBGs) was positioned at the same axis, but outside of the road.

For a given measurement, the outputs of all the 6 FBG strain sensors were acquired simultaneously on the input channel of the interrogator that was located in a remote location. A commercial spectrometer-based interrogator with an acquisition rate of 1 kHz, an absolute wavelength measurement accuracy of ±40 pm, and a precision of ±1 pm was used in the measurements. The interrogator unit was connected to a PC for further signal processing and storage of the data.

### 2.1. Fabrication of Fiber Bragg Gratings

**Figure 2:**
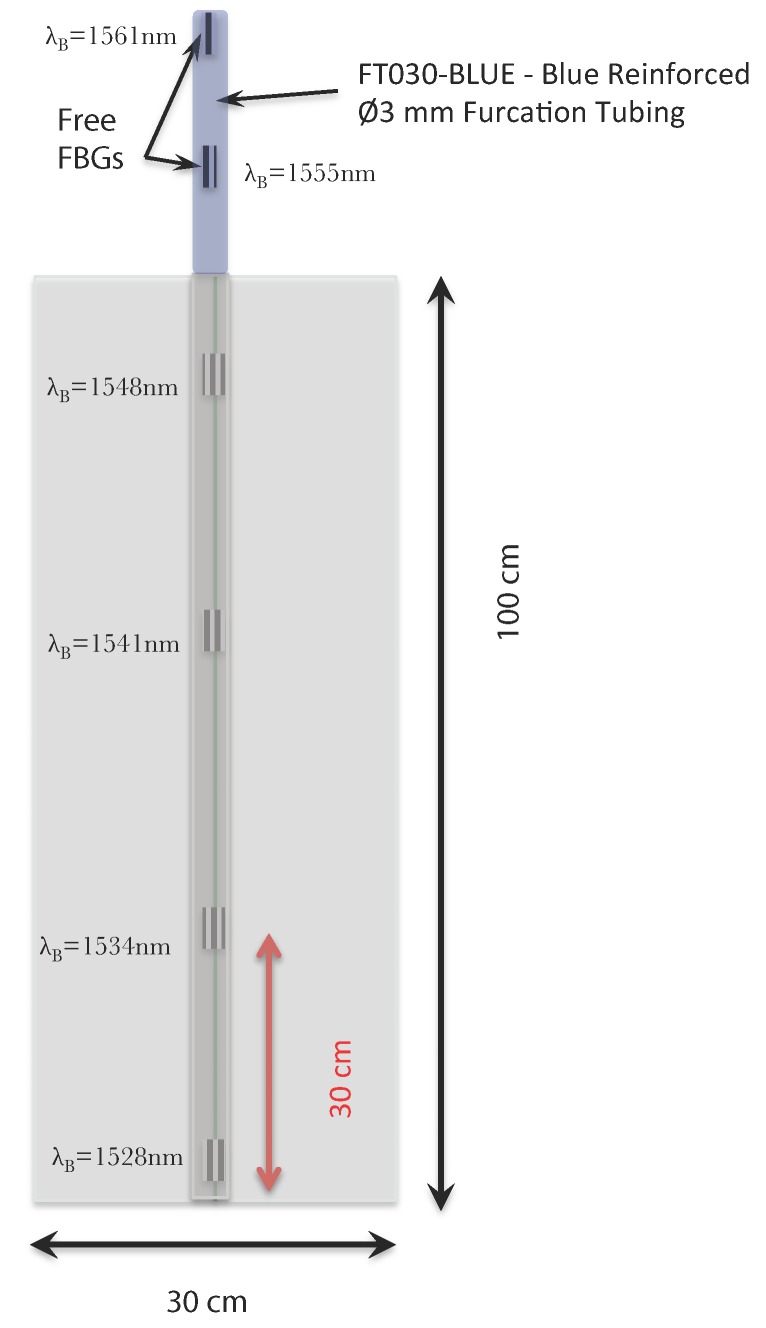
General view of the sensor pad.

The FBGs used in our implementation have been inscribed by using a continuous wave laser (AzurLightSystem–Sirah laser system) emitting at 244 nm. The method of inscription was based on the Lloyd mirror technique, allowing us to obtain a chain of 6 FBGs at different wavelengths equally distributed along the optical fiber. Four FBGs were glued in the sensing part (the platform), and the two others were left free outside of the sensing region. The length of each FBG was 3 mm, and the reflectivity was around 50%. Figure 2 presents a general view of the sensor pad with the position of the FBGs and the dimension of this platform.

### 2.2. Design of the Sensor Platform

**Figure 3:**
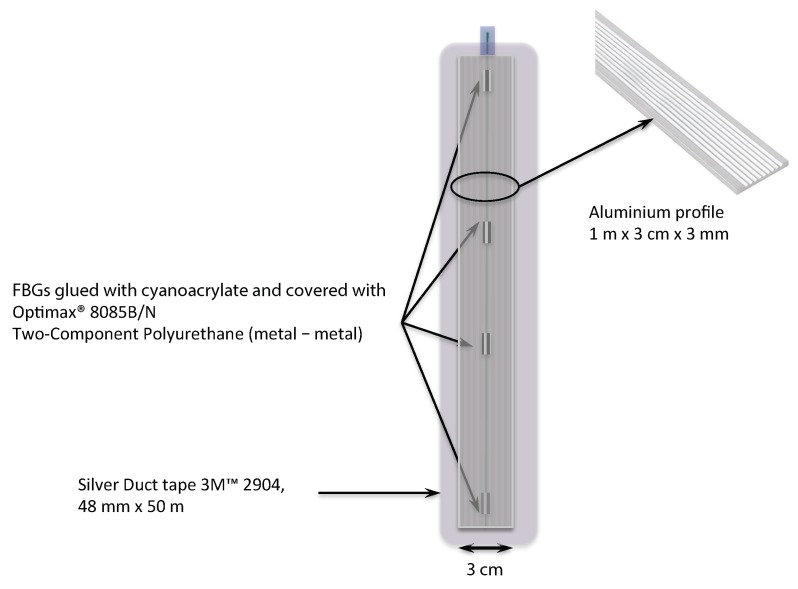
Zoom of the sensor part of the platform (upper view).

The overall dimensions of the platform allowed for good stability and contact with the soil without being too bulky. This facilitated its transport and positioning on the road without disrupting the traffic or having consequences on the vehicles. Figure 3 is a zoom of the active sensor part with the position of the FBGs. These FBGs were glued inside a groove of an aluminum profile. First of all, the FBGs were fixed in this groove with a small strain (150 µε) using a high-speed curing cyanoacrylate glue and then, a strong two-component glue was used to maintain the optical fiber all along the inside the groove. The same glue was used to fix a second aluminum profile over the first one and therefore to encapsulate the optical fiber, thus ensuring a good protection. Finally, a strong duct tape was used to perfectly maintain the active sensor part on the flat steel plate.

**Figure 4:**
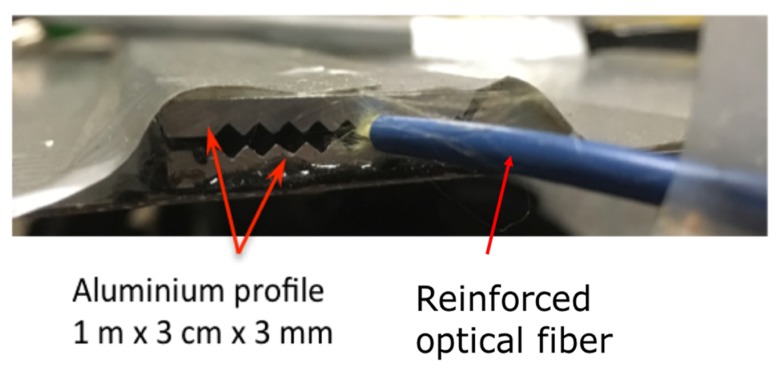
Picture of the ingress/egress of the optical fiber.

**Figure 5:**
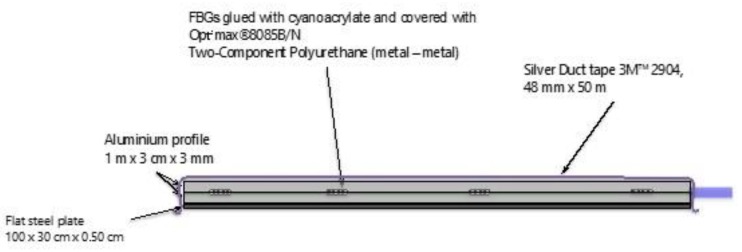
Zoom of the sensor part of the platform (lateral view).

Figure 4 is a picture of the egress/ingress of the optical fiber. Figure 5 is a schematic representation of the lateral view of the active sensor part with the main components.

**Figure 6:**
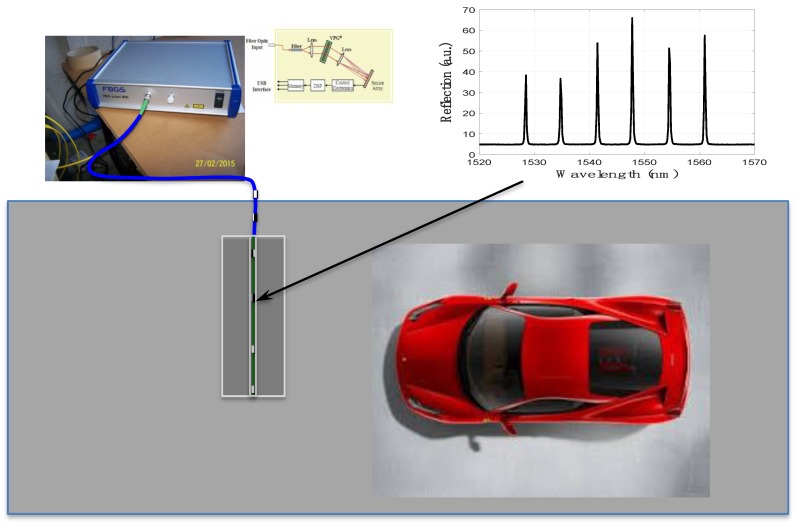
Schematic representation of the setup.

Figure 6 presents a global view of the measurement set-up. We can see the spectra of the FBGs (upper right) obtained with a FBG-Scan 800 interrogator. The measurement platform is suitable to be placed directly on the traffic lane due to its thickness (~3 cm) without affecting vehicle speeds or greatly reducing engineering difficulties.

During the first measurement campaign, the measurement platform was positioned on a side road in Brussels during normal operation (typical car speed at the measurement point was about 30–40 km h^−1^) to measure the speeds of cars. During a time span of 4 h, more than 50 cars were detected and their pictures were taken to determine the car types.

## 3. Measurement Results

When a strain is applied on the measurement platform due to the passage of a vehicle, a corresponding axial strain profile is created on the gratings, which can be demodulated by measuring the Bragg wavelength shifts.

**Figure 7:**
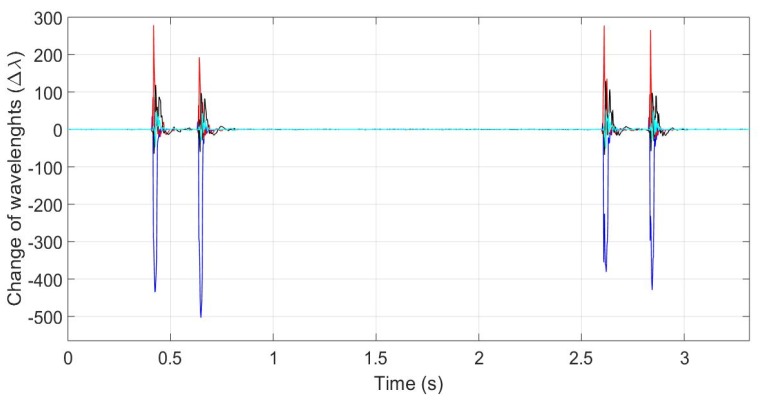
Traces of two successive cars creating 4 peaks corresponding to 4 wheels. There is an interval of about 2 s between the two cars. Different colors represent the responses of 4 FBGs (Fiber Bragg Gratings).

The typical traces measured by using the four FBG strain sensors inside the platform are shown in Figure 7 (the different colors represent the responses of the four FBGs). The passage of two successive cars with an interval of about two seconds between them can be observed in Figure 7. The amount of wavelength shift varies between the FBGs, depending on the position of the wheels.

**Figure 8:**
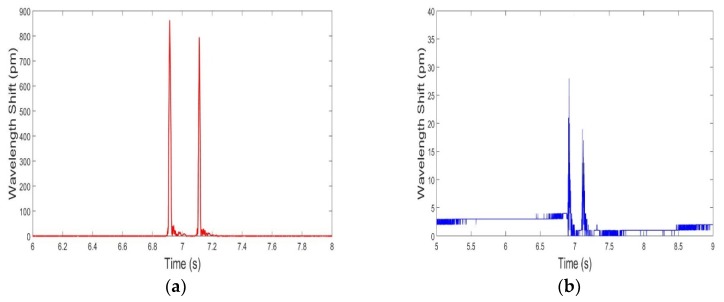
Amplitude of the wavelength shift (for a passage of the same car, circulating at 45 km h^−1^): (**a**) of FBG 2 (placed on the road); (**b**) of FBG 5 (outside of the road). A factor of 32 was measured between two amplitudes (wavelength shifts).

From a general point of view, FBG sensors are not required to be specifically placed on the road for counting axles or for speed calculation. In most of the applications presented so far in the literature, sensors have been placed just aside from the road or the railway. The FBG sensors placed on the road can provide additional information about the weight of the vehicle in motion. Indeed, the Bragg wavelength shift is related to the strain induced by the weight of the vehicle exerted on the wheels in transit. This phenomenon is represented in Figure 8, where one can compare the Bragg wavelength shifts of the FBG sensors on the road (Figure 8a) with the shifts of those outside of the road (Figure 8b). The FBGs outside of the road would be useful in compensating temperature changes (between day and night or between seasons).

During the first group of the measurements, the speed values of tens of cars were measured. The speed of vehicles was calculated as:

$$v=\frac{d_{axle}}{\Delta t}3.6\;\left[km/h\right]$$

where Δt represents the time difference between the passage of the wheels (e.g., two peaks on Figure 8a) and $d_{axle}$ represents the distance between the axles of the vehicle ($d_{axle}$ is taken as 2.5 m in our calculations as the car type is not known a priori).

Due to our long-term outdoor installation, numerous field tests were carried out (with intervals of a few days) to validate both the hardware of the monitoring system and the related signal demodulation algorithm. The results demonstrate the capability of the proposed system in both the detection of the vehicle and its speed estimation. During the tests, the speed of tens of cars was measured, and 10 different vehicle models were observed. Table 1 summarizes some examples. Error values in Table 1 were determined taking ±0.5 m of tolerance on the $d_{axle}$ of 2.5 m used for speed calculations.

With the ultimate goal of the research being to develop a weigh-in-motion system, a second group of measurements was realized on a road closed to public traffic. In practice, it is hard to ensure the vehicle always drives in the same path, which affects the accuracy of further signal processing steps such as weight-in-motion measurements.

**Figure 9:**
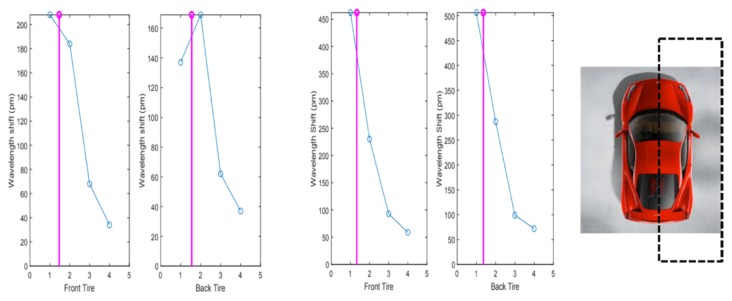
Example cases of the wheel tracing process based on the wavelength shift induced on the four cascaded FBGs (the straight line represents the position followed by the driver).

In this part of the experiment, the position of the wheels was successfully traced by comparing the wavelength shifts of four FBG sensors in the platform. As the test drives were organized along straight lines, the front and back tires of the car followed the same trajectory as shown in Figure 9 for two example cases. For each example case, two graphs are given (for the front tire and back tire of the same vehicle and same passage), resulting in four data plots in Figure 9. The integer numbers in the horizontal axis of the graphs represent the number of the FBG in the array (from 1 to 4). When the vehicle passed through the platform, the FBG nearest to the tire position was subject to more strain compared to the others (hence presenting more wavelength shift). Therefore, measuring and plotting the wavelength shifts of the FBGs in this array provided us with information about many aspects of the vehicle’s passage through the platform, such as the tire’s passage position, direction change, and the load distribution of the vehicle.

The measurement platform covered the half of the car as represented by the dashed line on the right-hand side of Figure 9.

**Figure 10:**
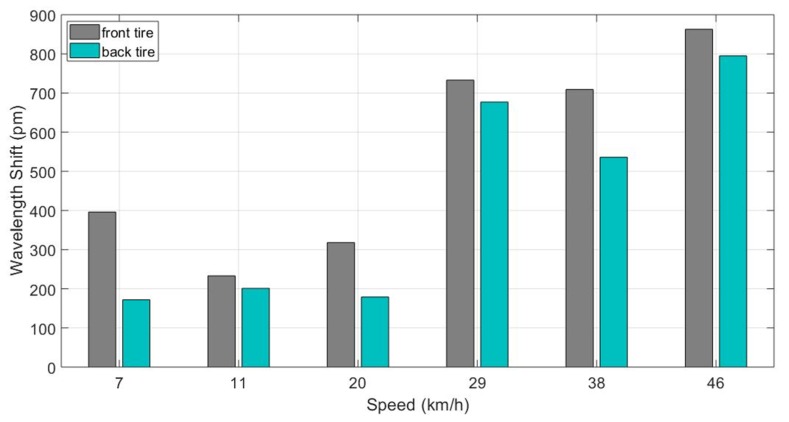
Wavelength shift amplitude for 6 different speed values (front and back tires).

In the final stage of the measurement, several tests were done with the same car passing at the same position with various known speeds. The goal of these tests was to determine the correlation between the speed and the magnitude of the peak wavelength shifts. Figure 10 summarizes the preliminary results for six different speed values. For speed values higher than 20 km h^−1^, the wavelength shift amplitude considerably increased. The shift in amplitude was always higher for the front tire of the car compared to its back tire. This observation is in agreement with the other experimental work reported in the literature [8] and can be explained by the fact that the weight of the front is always heavier (because of the engine) than that of the rear for a front-wheel-drive vehicle.

The intermediate conclusion based on this experiment is that the information of car speed should be used as a part of the calibration factor for calculating the weight of the vehicle in motion. Further tests are required to determine such calibration parameters as a vehicle’s dynamic weight can be influenced by many factors such as speed, acceleration, tire pressure, and pavement conditions [9]. Once the calibration is realized, the static load can be estimated from the measured dynamic load and calibration parameters can be applied.

**Figure 11:**
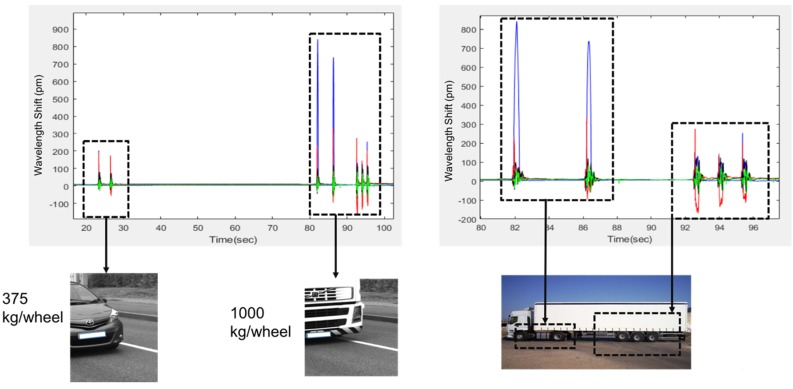
Roadmap for weigh-in-motion determination.

With the goal of designing a weigh-in-motion system, some special configurations of vehicle passages were analyzed. The details of an example case are represented in Figure 11, in which a five-axle empty truck passes about 1 min after a two-axle car. The amplitude of the wavelength shift was observed to be proportional to the actual total weight of these two vehicles. The distribution of the weight of the truck between the first two axles and the last three axles was clearly observed in the same figure. However, within this study, a quantitative conclusion cannot be made for the relationship between the speed of car and the amplitude of the wavelength shift.

## 4. Conclusions

Realizing field tests concerning the application of Fiber Bragg Grating sensors is of crucial importance for the monitoring of both railways [10,11] and roads [12,13,14]. Even though research regarding fiber-based weigh-in-motion systems has been reported over the last 20 years, none of these references offer a quantitative analysis about weight measurements in the form of a final product that conforms to transportation regulations [15,16]. Exploiting already-installed fibers for distributed sensor applications has been attracting great interest [17,18]. Particularly, the co-deployment of fiber optic cables along transport infrastructure (including railways and roads/highways) provides very suitable means for such applications and can be considered as an alternative approach to custom-made traffic sensor platforms.

In this work, a mobile measurement platform containing cascaded FBGs was developed. The results obtained during a measurement campaign carried out on a side road in Mons (Belgium) showed that our mobile measurement platform is an effective method to detect vehicles and measure their speed during normal traffic flow. The whole experimental work proved the robustness of the platform and a good response of the sensor. The preliminary qualitative results obtained on vehicles of different types and weights show that this platform, after calibration, could be well adapted for dynamic weight measurements. Moreover, through experiments with known speeds and paths, more insight has been obtained about the relationship between the velocity and the position of the vehicle and the response of the FBG sensor.

## Figures and Tables

**Table 1:** Speed values measured by the proposed FBG platform for various car types under normal traffic conditions.

	Measured Speed (km h^−1^)	Error (±)
model 1	33.58	6.71
model 2	37.50	7.50
model 3	40.50	8.20
model 4	34.06	6.90
model 5	30.82	6.16
model 6	25.50	5.00
model 7	40.18	8.03
model 8	24.35	4.86
model 9	30.60	6.12
model 10	28.10	7.60
